# Lymphoblast Oxidative Stress Genes as Potential Biomarkers of Disease Severity and Drug Effect in Friedreich's Ataxia

**DOI:** 10.1371/journal.pone.0153574

**Published:** 2016-04-14

**Authors:** Genki Hayashi, Gino Cortopassi

**Affiliations:** Department of Molecular Biosciences, University of California Davis, Davis, California, United States of America; Northwestern University Feinberg School of Medicine, UNITED STATES

## Abstract

There is no current approved therapy for the ultimately lethal neuro- and cardio-degenerative disease Friedreich's ataxia (FA). Finding minimally-invasive molecular biomarkers of disease progression and drug effect could support smaller, shorter clinical trials. Since we and others have noted a deficient oxidative stress response in FA, we investigated the expression of 84 genes involved in oxidative stress, signaling, and protection in control and FA lymphoblasts ranging from 460 to 1122 GAA repeats. Several antioxidant genes responded in a dose-dependent manner to frataxin expression at the mRNA and protein levels, which is inversely correlated with disease progression and severity. We tested the effect of experimental Friedreich’s ataxia therapies dimethyl fumarate (DMF) and type 1 histone deacetylase inhibitor (HDACi) on biomarker mRNA expression. We observed that exposure of lymphoblasts to DMF and HDACi dose-dependently unsilenced frataxin expression and restored the potential biomarkers NCF2 and PDLIM1 expression to control levels. We suggest that in addition to frataxin expression, blood lymphoblast levels of NCF2 and PDLIM1 could be useful biomarkers for disease progression and drug effect in future clinical trials of Friedreich’s ataxia.

## Introduction

Friedreich’s Ataxia (FA) affects 1 in 40,000 individuals and is considered the most common autosomal recessive ataxia[1]. Patients suffer from vision and hearing loss, gait ataxia reducing motor coordination, and weakness and atrophy of the extremities[2, 3]. The pathology of the disease is characterized by the neurodegeneration of the cerebellar tissue and demyelination in spinocerebellar dorsal root ganglion neurons as well as hypertrophic cardiomyopathy and diabetes[4–6]. Friedreich’s ataxia is most commonly caused by trinucleotide repeat expansion of GAA within the first intron of the nuclear encoded gene frataxin, leading to reduced expression by gene silencing[1, 7–10].

Growing evidence suggests that oxidative stress is involved in the pathogenesis of FA. It is known that frataxin enhances the biosynthesis of iron-sulfur clusters that in turn bind to mitochondrial complexes and aconitase in order to promote the transfer of iron and sulfur during synthesis[11–14]. While the mechanism that causes elevated reactive oxygen species (ROS) in FA remains unclear, iron-sulfur cluster deficiency is thought to reduce thiol and aconitase dependent oxidative stress protection[15, 16]. The importance of ROS and oxidative stress sensitivity in FA has been implicated since the causal mutation of FA was identified. Studies by Emond et al. and Schulz et al. in 2000 have shown that patients had increased levels of blood plasma dihydroxybenzoic acid, malondialdehyde, and urine 8-hydroxy-2'-deoxyguanosin, all of which are markers of ROS[17, 18]. In 2010, Haugen et al. identified significantly increased nuclear and mitochondrial DNA lesion formation in FA patients[19]. FA patient-derived cells have been assessed for their sensitivity to oxidative stress in response to external stimuli. In 2001, Chantrel-Groussard et al observed reduced superoxide dismutase induction in patient cells treated with oligomycin; this drug is associated with thiol mediated defense against ROS, which leads to cell death[20]. Additionally, in 1999, Wong et al showed that patient cells were more sensitive to hydrogen peroxide treatment, which was abated in the presence of iron/calcium chelators and apoptosis inhibitors[21].

Furthermore, a reduction in oxidative stress responses has been indicated in the pathogenesis of FA[22]. In 2013 and 2014, our previous work and Sandi et al showed that two related frataxin deficient transgenic mice harboring human genes had significant reduction in basal expression of major antioxidants, notably Glrx1, Gstm1, Gpx1, Hmox1, Nqo1, Prdx3, Sod2 and Txnrd. Many of these genes are Nrf2 regulated to reestablish cellular redox homeostasis[23, 24]. Nrf2, a major regulator of oxidative stress response, is also known to be dysregulated in FA. Additionally, Nrf2 protein translocation is significantly reduced in both mouse models, patient-derived cells, and frataxin knockdown cells[23, 25, 26]. This leads to the idea that, while normal cells can react to and alleviate elevated levels of ROS, frataxin deficient cells are unable to cope with the insult due to a dysfunctional oxidative stress response. Additionally, Abeti et al, 2015 has shown that Nrf2 inducers can reduce cell death and lipid peroxidation induced by ROS in transgenic mice harboring human genes[27].

FA patients currently have no treatment options, and methods to test the efficacy of possible therapeutics are limited. Current aims for the treatment of FA include directly targeting and restoring frataxin expression or targeting the downstream oxidative stress response pathway effects associated with FA. As not all treatments for FA directly target frataxin expression, we decided to investigate the expression of oxidative stress response genes as biomarkers of FA. We hypothesized that the expression of select oxidative stress response genes is altered in FA patients and that these changes are correlated with relative frataxin expression. The use of evaluating biomarker expression in conjunction with frataxin expression is potentially useful to assess drug therapies that aim to specifically restore frataxin expression, such as the histone deacetylase inhibitor (HDACi) RGFP109/RG2833[28] and the Nrf2 inducer dimethyl fumarate (DMF)[29]. In addition, these newly discovered biomarkers might also indicate if compounds such as interferon gamma-1b[30] or the antioxidant EPI-743[31] that are currently in clinical trial for FA can reverse the downstream pathomechanism of frataxin deficiency, even if they do not alter frataxin expression directly.

We first performed a *primary* qPCR array screen to identify ten candidate genes out of 84 that had altered expression in FA-derived b-lymphoblast cells compared to control cells, i.e. 84 biomarkers → 10. We then tested these ten candidate genes in a *secondary* qPCR screen utilizing in-house primers, and verified the expression changes of six out of ten gene candidates, i.e. 10 biomarkers → 6. In a *tertiary* screening, we evaluated protein expression changes in FA patient-derived b-lymphoblasts of the six candidate genes identified in the secondary qPCR analysis screen. The tertiary screen identified four genes that showed similarly altered protein and mRNA expression, i.e. 6 biomarkers → 4. These four candidate biomarkers were then tested for their mRNA response to two possible FA therapies that are able to induce frataxin expression: the previously identified Nrf2 inducer DMF[29], and a type 1 HDACi RGFP109/RG2833[32, 33]. Two of the four biomarkers, NCF2 and PDLIM1, proved responsive to two Friedreich's therapeutic molecules with demonstrated activity to unsilence frataxin, i.e. 4 biomarkers → 2, NCF2 and PDLIM1.

Our work demonstrates that the mRNA abundance of the oxidative stress response genes NCF2 and PDLIM1 are potential biomarkers for FA. We suggest that NCF2 and PDLIM1 report on oxidative status in Friedreich's lymphoblasts. Both NCF2 and PDLIM1 have a dose-dependent correlation with relative frataxin expression that is ameliorated by treatment of frataxin inducers. The identification of non-frataxin biomarkers of disease severity and drug effect (NCF2 and PDLIM1) in available blood lymphocytes could support the clinical testing of drugs that do not raise frataxin as their primary consequence, but improve oxidative status downstream of frataxin deficiency. Additionally, because NCF2 and PDLIM1 respond dose-dependently to frataxin level, they supplement and confirm the antioxidant effects of therapies that raise frataxin, increasing the number of Friedreich's relevant biomarkers to three in the lymphoblast context: frataxin, NCF2, and PDLIM1. Lastly, because PDLIM1/CLP36 has recently been shown to be responsive to Nrf2 status/oxidative stress in cardiomyocytes, it could become a valuable marker of drug effect in cardiomyocytes.

## Material and Methods

### Cell culture

Friedreich’s ataxia patient-derived b-lymphoblasts: GM16220, GM16216, GM16214, GM15850, GM14518, p614, p545 and p75, and healthy control b-lymphoblasts: GM13068, GM09869, GM03715, GM02184, GM02131, GM00607, and GM00333 (Coriell Institute, Camden, NJ and kind gift from Dr. Franco Taroni, Milan, Italy) were maintained at 37°C in a humidified atmosphere with 5% CO_2_. DMEM (Corning, inc., Corning, NY) supplemented with 15% fetal bovine serum (JR-Scientific, Woodland, CA), 2mM sodium pyruvate (Sigma-Aldrich, St. Louis, MO), 1x Penicillin-Streptomycin Solution (Corning, inc., Corning, NY), 1x MEM non-essential amino acids (Invitrogen, Carlsbad, CA), and 50 mg/ml uridine (MP-Biomedicals, Solon, OH) was used to grow cells. Media was changed every two days.

### Drug treatment of b-lymphoblasts

The b-lymphoblasts were initially seeded in six well plate format at 0.3x10^6^ cells per well. The cells were incubated with 0.1% DMSO (Sigma-Aldrich, St. Louis, MO) as vehicle control or 3–30μM of dimethyl fumarate (Sigma-Aldrich, St. Louis, MO) or HDACi (MedChem Express, Monmouth Junction, NJ) dissolved in DMSO. Following a 48 hour incubation period, total mRNA or protein was extracted per methods described below.

### RNA extraction and quantitative RT-PCR

Total mRNA was extracted from b-lymphoblast cells using RNeasy plus mini kit (Qiagen, Valencia, CA) following manufacturer’s instruction and mRNA quantity and quality was measured by NanoDrop 2000c Spectrophotometer (Thermo Scientific, Waltham, MA).

cDNA was synthesized from mRNA with iScript cDNA Synthesis Kit (Bio-Rad, Hercules, California) in C1000 Touch Thermal Cycler (Bio-Rad, Hercules, California) per manufacturer’s instruction. SensiFAST SYBR No-ROX Kit (Bioline, Taunton, USA) was used to perform qPCR on the synthesized cDNA in a Roche Lightcycler 480 (Roche Diagnostics, Indianapolis, IN). The second derivative of the amplification curve was used to determine the cycle threshold, and the data were analyzed by delta-delta CT calculation[34]. Primer sets used in qPCR are listed in Table 1.

**Table 1:** List of qPCR primer sets for biomarker candidates in the secondary qPCR screening.

Gene	Sequence (5' → 3')
DUSP1 Forward	GTACTAGCGTCCCTGACAGC
DUSP1 Reverse	GGCCACCCTGATCGTAGAG
NCF2 Forward	GGTGCCCCTTTCAGAAGACA
NCF2 Reverse	AAAGCCTTGGTCACCCACTG
PDLIM1 Forward	GAGTTGAATGAGCCCCCGAA
PDLIM1 Reverse	AATCCTGAGGGCTTGTTGGG
PRDX2 Forward	CGAGCATGGGGAAGTTTGTC
PRDX2 Reverse	GGCACAAGCTCACTATCCGT
PRDX5 Forward	TTCAAGGGCAAGAAGGGTGTG
PRDX5 Reverse	GGCAGGTGTGTCTTGGAACAT
SFTPD Forward	TGAAGGGGGACAAAGGCATTC
SFTPD Reverse	AGAAGCAACATCTGGAAGCCC

### Primary screening quantitative RT-PCR

Total mRNA was extracted as previously described. Genomic DNA was eliminated, and cDNA was synthesized with RT2 First Strand Kit (catalog 330401). 5ug of total mRNA was treated with buffer GE and incubated for 5 min at 42°C, then placed immediately on ice for at least 1 min. Following the addition of reverse-transcription mix, the mRNA was incubated at 42°C for 15 min then at 95°C for 5 min. The cDNA was diluted 1:10 and added to RT2 SYBR Green qPCR Mastermix (catalog 330502). The cDNA-Mastermix was aliquoted into RT2 Profiler PCR Array Human Oxidative Stress & Antioxidant Defense plate (catalog PAHS-065ZF-12) with 50ug of cDNA added to individual wells of the 96 well plate. qPCR was performed on Roche Lightcycler 480 (Roche Diagnostics, Indianapolis, IN) with initial 10min incubation at 95°C followed by 45 cycles of 95°C for 15sec and 60°C for 1min. The second derivative of the amplification curve was used to determine the cycle threshold, and the data were analyzed by delta-delta CT calculation[34].

### Protein extraction and western blot analysis

Human b-lymphoblast cell pellets were homogenized with a cell lysis buffer containing Protease/Phosphatase Inhibitor Cocktail (Cell Signaling, Danvers, MA) and PMSF (Sigma-Aldrich, St. Louis, MO). Mixtures of 20ug total protein lysate and 50mM DTT were loaded into 4%–12% Bis–Tris gels (Invitrogen, Carlsbad, CA). Electrophoresis was carried out according to the manufacturer’s recommendations. Following electrophoresis, the proteins were transferred to nitrocellulose membranes by the Trans-Blot Turbo Transfer System (Bio-Rad, Hercules, California) per manufacturer’s instruction and blocked with an Odyssey TBST blocking buffer (LI-COR Biosciences, Lincoln, NE) for 1 hour. Membranes were incubated overnight with the following primary antibodies in blocking buffer: β-tubulin (DSHB-E7; DSHB, Iowa), Frataxin (sc-25820 Santa Cruz Biotechnology), NCF2, PDLIM1, SFTPD (ab109523, ab126628, ab15696 respectively, Abcam, Cambridge, MA) and PRDX5 (LF-MA00017 Abfrontier, Seoul, Korea). The membranes were washed three times with TBS-Tween (EDM Millipore, Billerica, MA) then incubated with anti-rabbit IRDye 680CW and anti-mouse IRDye 800CW-coupled secondary antibodies (LI-COR Biosciences, Lincoln, NE) for 1 hour. Fluorescence was visualized with the Odyssey infrared imager and software (LI-COR) according the manufacturer’s instruction.

### Data analysis

Data analysis was carried out with GraphPad Prism 5.0 statistics software (GraphPad Software, La Jolla, CA). The list of analyses include: Two-way ANOVA with Bonferroni post-hoc multiple comparison test, One-way ANOVA with Newman–Keuls post-hoc multiple comparison test, correlation, and linear regression.

## Results

### Primary qPCR array screening of altered oxidative stress response biomarkers

**Fig 1:**
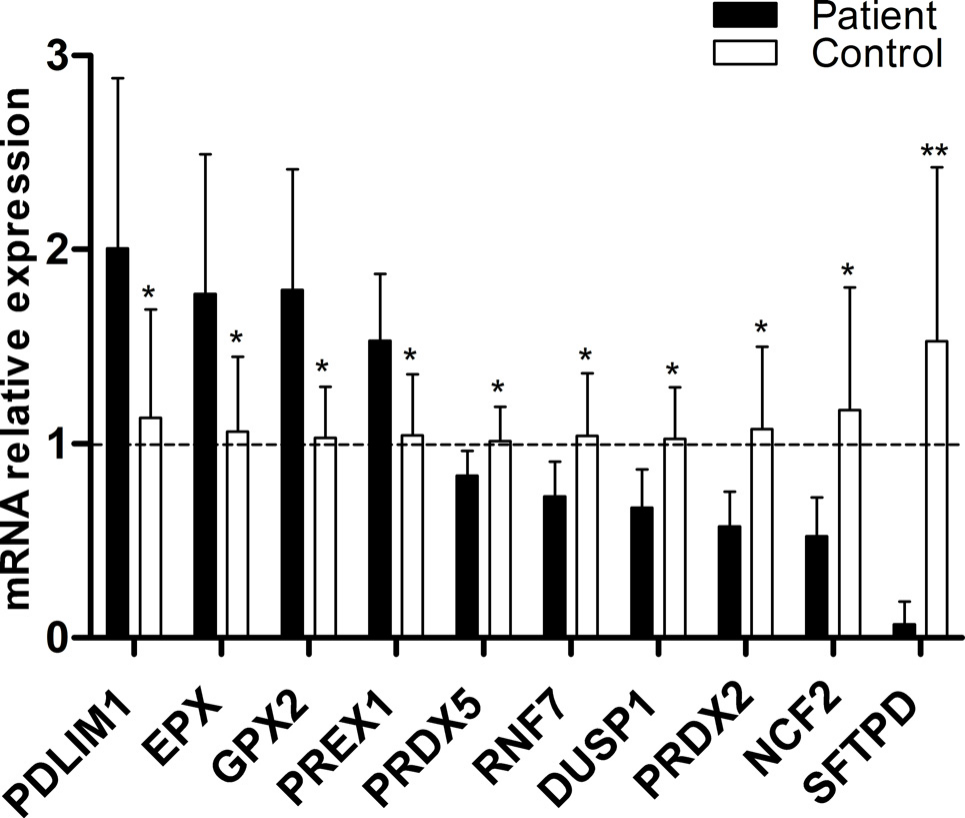
Primary screening of potential Friedreich’s ataxia mRNA biomarkers by Qiagen’s human oxidative stress and antioxidant defense qPCR array. Relative expression was normalized to the average of β-actin, GAPDH, HPRT1 and RPLP0 using ΔΔ^CT^ calculation. The mRNA expression of 84 genes involved in oxidative stress response was compared between FA and healthy control b-lymphoblast cells. Of the 84 candidate genes, ten were significantly altered: PDLIM1(1.77 fold), EPX (1.67 fold), GPX2 (1.73 fold), PREX1 (1.47 fold), PRDX5 (0.82 fold), RNF7 (0.70 fold), DUSP1 (0.65 fold), PRDX2 (0.53 fold), NCF2 (0.45 fold) and SFTPD (0.044 fold). Bars represent averages±standard deviations (n = 7, p<0.05*, p<0.01**, p<0.001***). Data in S1 File.

Using the human oxidative stress and antioxidant defense qPCR array, mRNA expression was analyzed by qPCR for eight Friedreich’s ataxia patient and seven healthy control b-lymphoblast cells. Of the 84 genes available on the qPCR array, ten genes; PDLIM1 (p<0.039, n = 7), EPX (p<0.034, n = 7), GPX2 (p<0.011, n = 7), PREX1 (p<0.014, n = 7), PRDX5 (p<0.042, n = 7), RNF7 (p<0.049, n = 7), DUSP1 (p<0.014, n = 7), PRDX2 (p<0.020, n = 7), NCF2 (p<0.036, n = 7), and SFTPD (p<0.0050, n = 7) showed significantly altered expression as compared to healthy control (Fig 1). Of the ten potential biomarkers, four were significantly overexpressed while six were significantly underexpressed, ranging from 2.00 to 0.068 fold change relative to healthy control b-lymphoblasts.

### Secondary qPCR analysis screening to identify oxidative stress response biomarkers

**Fig 2:**
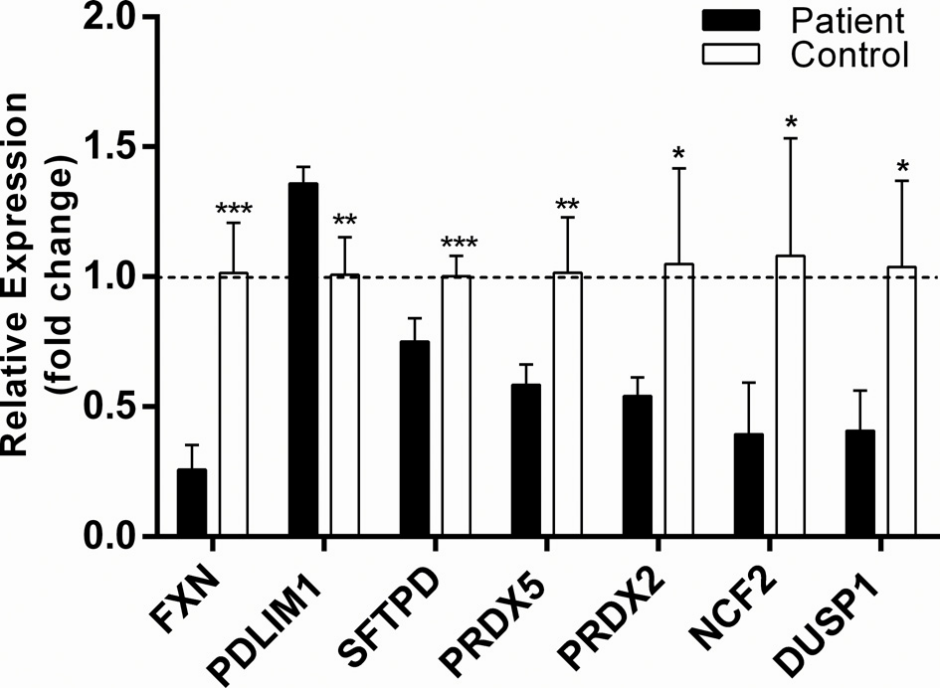
Secondary qPCR screening of potential Friedreich’s ataxia mRNA biomarkers by qPCR using in-house primers. Relative mRNA expression of frataxin as well as the ten potential biomarkers identified in the primary screen were normalized to β-actin using the ΔΔ^CT^ calculation. Relative frataxin expression in the FA cells was 0.25 fold of healthy control b-lymphoblasts. In the same samples, six of the ten potential biomarkers were consistent with the primary screening results: PDLIM1 (1.34 fold), SFTPD (0.78 fold), PRDX5 (0.58 fold), PRDX2 (0.52 fold), NCF2 (0.37 fold), and DUSP1 (0.39 fold). Bars represent averages±standard deviations (n = 4, p<0.05*, p<0.01**, p<0.001***). Data in S2 File.

As a secondary screening of biomarker candidates, we utilized qPCR to measure the mRNA expression of the ten genes that were significantly altered in the primary Qiagen screening array of FA patient-derived cells compared to control b-lymphoblasts. On average, frataxin mRNA expression in the patient b-lymphoblast cell lines was 0.25 fold of the healthy control cells (p<0.00043). Of the ten primary hits, six were significantly altered in the secondary qPCR screen: PDLIM1 (1.34 fold, p<0.0045, n = 4), SFTPD (0.77 fold, p<0.0055, n = 4), PRDX5 (0.57 fold, p<0.0090, n = 4), PRDX2 (0.51 fold, p<0.035, n = 4), NCF2 (0.36 fold, p<0.032, n = 4), and DUSP1 (0.39 fold, p<0.014, n = 4) (Fig 2). Of the genes tested, EPX, GPX2 and PREX1 were not reproducible from the primary screening.

**Fig 3:**
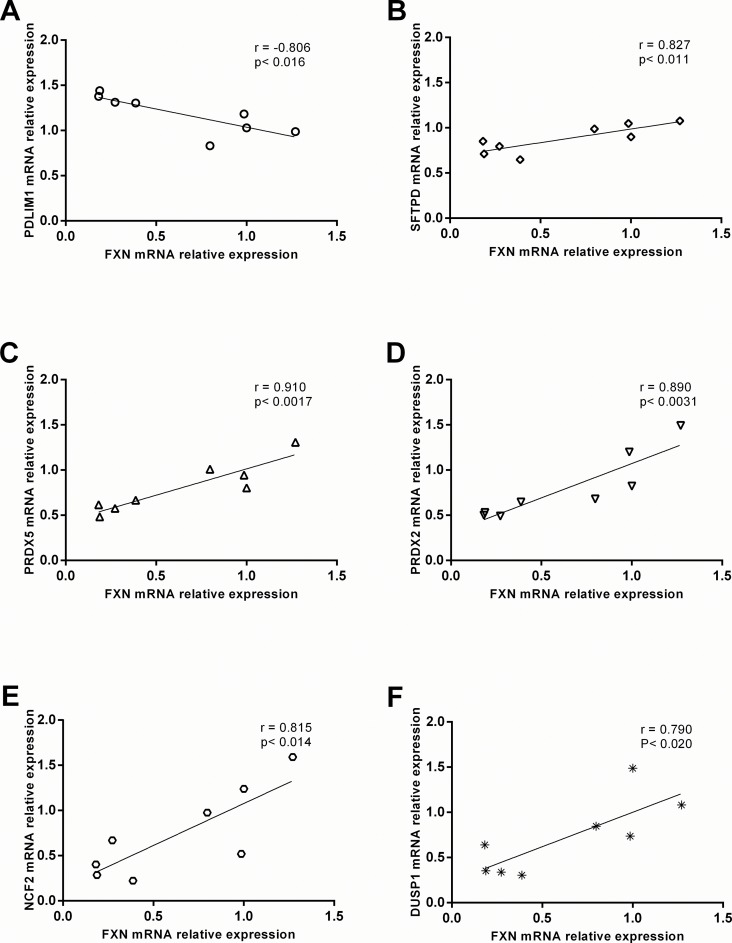
Correlative analysis of relative frataxin mRNA expression to biomarkers candidate expression in b-lymphoblasts. mRNA expression of candidate biomarkers is represented on the y-axis while frataxin expression is represented on the x-axis. Correlation was identified as strong if the coefficient is ±0.8 and somewhat strong at ±0.7 (n = 8). Data in S2 File.

Correlative analysis between relative mRNA expression of candidate biomarkers and frataxin was also evaluated in order to understand the dependency of biomarker expression on frataxin levels. Of the biomarkers that were significantly correlated with relative frataxin expression, six showed a strong to somewhat strong positive correlation coefficient ranging from 0.79 to 0.91, while one biomarker had a strong negative correlation coefficient of -0.806 (p<0.020, n = 8) (Fig 3). Healthy control data was removed from the correlative analysis to prevent bimodal frataxin expression driving the correlation. Taken together, the results support the identification of six frataxin-dependent oxidative stress response pathway biomarkers: PDLIM1, SFTPD, PRDX5, PRDX2, NCF2, and DUSP1.

### Tertiary western blot analysis to identify candidate oxidative stress response biomarkers

**Fig 4:**
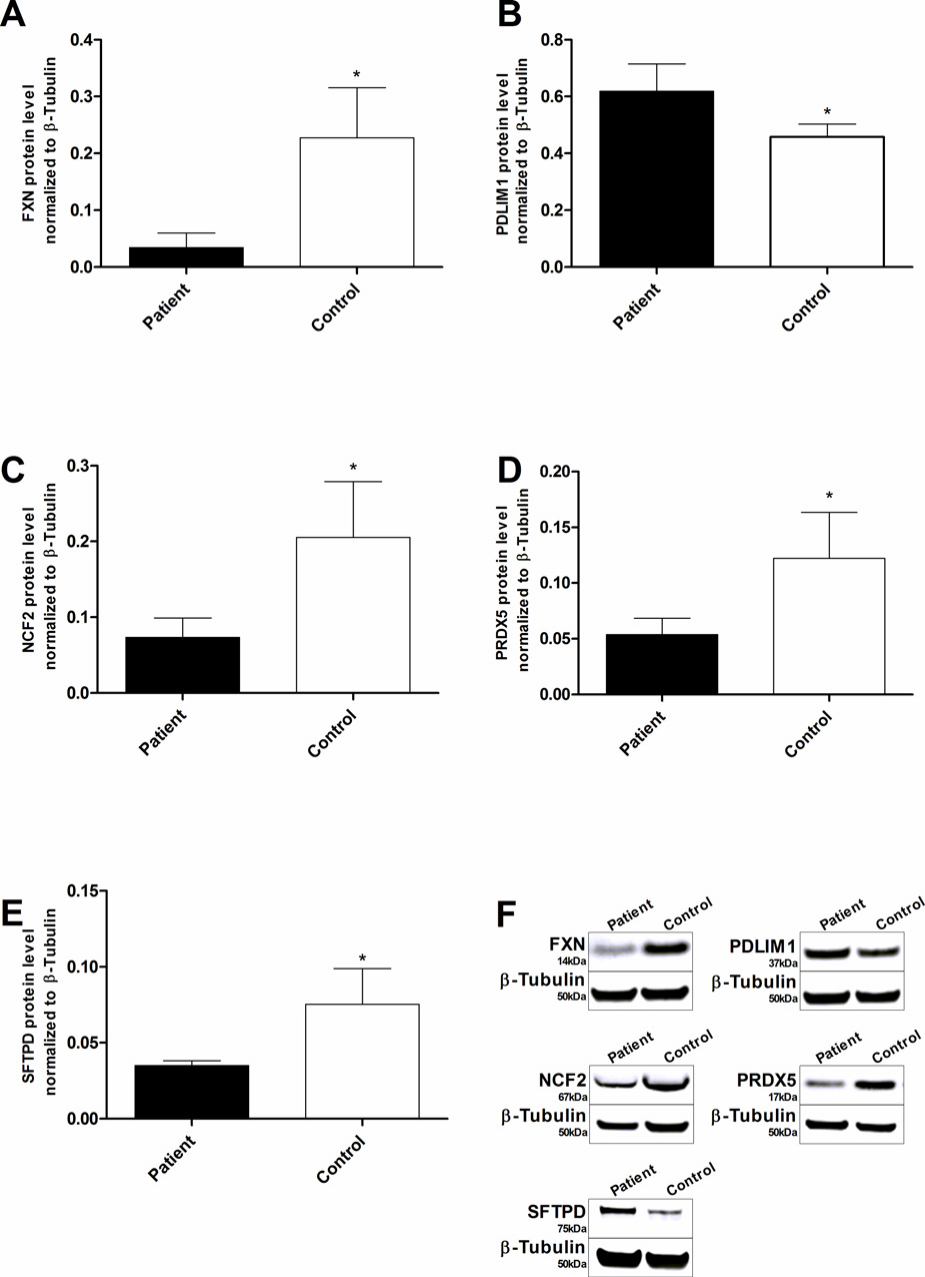
Tertiary screening of potential Friedreich’s ataxia protein level biomarkers by western blot. The target western blot fluorescence signals were normalized to β-Tubulin. The frataxin expression in the FA cell was 0.15 fold of the healthy control b-lymphoblasts. On the protein level, four of the six biomarkers were consistent with the secondary qPCR screening: PDLIM1 (1.35 fold), NCF2 (0.36 fold), PRDX5 (0.44 fold) and SFTPD (0.47 fold). Bars represent averages±standard deviations (n = 4, p<0.05*, p<0.01**, p<0.001***). Data in S3 File.

In addition to analyzing candidate biomarker expression at the mRNA level, the expression of biomarker candidates in FA and healthy control b-lymphoblasts was also confirmed on the protein level by western blot analysis. The average protein level in FA cells was 0.15 fold of the healthy control b-lymphoblast cell line (p<0.018). Protein expression of those genes identified in the secondary qPCR screening was assessed, and four genes: PDLIM1 (1.35 fold, p<0.023, n = 4), NCF2 (0.36 fold, p<0.032, n = 4), PRDX5 (0.44 fold, p<0.039, n = 4) and SFTPD (0.47 fold, p<0.015, n = 4) were significantly altered in the patient-derived cells compared to healthy controls (Fig 4). The biomarker candidates: PRDX2 and DUSP1 were not reproducible in the tertiary protein level screen as compared to the secondary qPCR screen.

**Fig 5:**
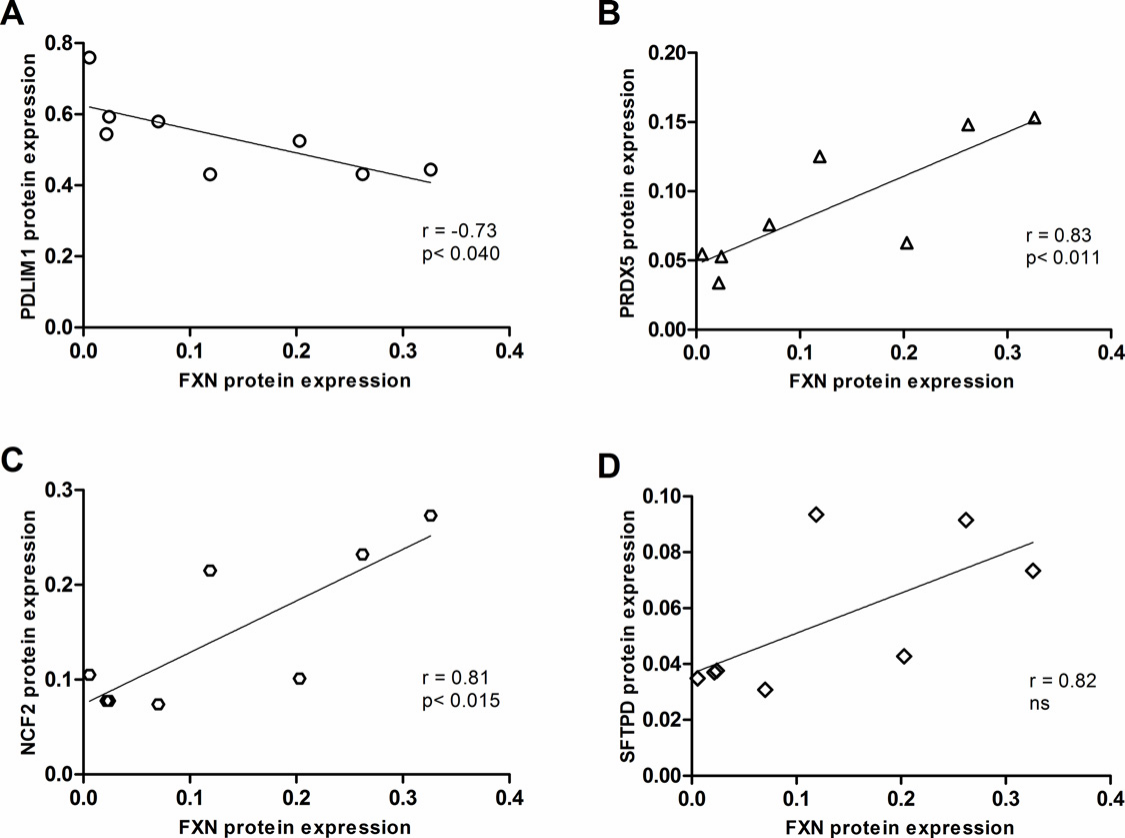
Correlative analysis of relative frataxin protein expression to biomarkers candidate expression in b-lymphoblasts. Biomarker expression is represented on the y-axis while the frataxin expression is represented on the x-axis. Correlation was identified as strong if the coefficient is ±0.8 and somewhat strong at ±0.7 (n = 8). Data in S3 File.

Of the four significantly altered protein level biomarkers, three showed significant correlation with frataxin protein expression. Two biomarkers: PRDX5 and NCF2 had a strong positive correlation coefficient ranging from 0.83 to 0.81 and one candidate: PDLIM1 had a somewhat strong negative correlation coefficient of -0.73 (p<0.040, n = 8) (Fig 5). Again, healthy control data was removed from the correlative analysis to prevent bimodal frataxin expression driving the correlation. Taken together, at the protein level, three oxidative stress response biomarker candidates: NCF2, PDLIM1 and PRDX5 are strongly to somewhat strongly dependent on frataxin expression.

Of the ten biomarker candidates identified in the primary qPCR screen, four biomarkers: PDLIM1, PRDX5, NCF2 and SFTPD were confirmed in the secondary qPCR and tertiary protein screen. The three screens were in agreement showing similar trend in the expression when comparing FA to healthy control b-lymphoblasts (Figs 2 and 4). Ultimately, frataxin level was more tightly correlated with the biomarker mRNA levels compared to protein levels, this result is likely due to the higher precision and accuracy of qPCR quantification compared to western blot procedures.

### Reversal of oxidative stress response markers in response to experimental Friedreich’s Ataxia therapies

#### Nrf2 stimulator (dimethyl fumarate)

To better assess the applicability of the identified biomarker candidates, we measured the dose-dependent change of the candidate biomarkers’ mRNA expression in response to two experimental Friedreich’s ataxia therapies: the Nrf2 stimulator DMF, and the type 1 HDACi RGFP109/RG2833.

**Fig 6:**
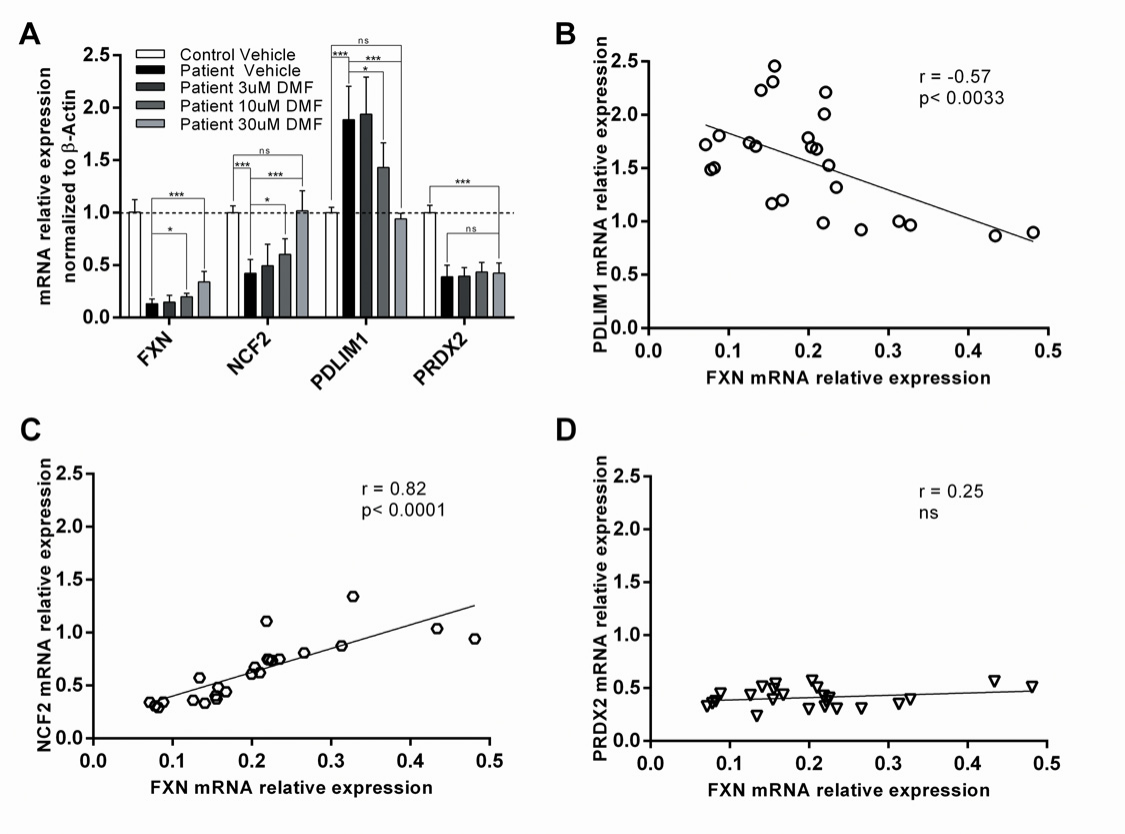
Friedreich’s ataxia biomarkers in response to 48 hours DMF treatment of patient b-lymphoblast as compared to healthy control. A) Relative mRNA expression was normalized to β-actin using ΔΔ^CT^ calculation. The average expression of frataxin at vehicle, 3μM, 10μM and 30μM DMF is 0.13, 0.15, 0.20 and 0.34 respectively in relative to healthy control b-lymphoblasts. In the same order, PDLIM expression is 1.89, 1.94, 1.43 and 0.94 on average, NCF2 expression is 0.42, 0.49, 0.60 and 1.02 on average, and PRDX2 is 0.39, 0.39, 0.43 and 0.42 on average relative to healthy control b-lymphoblasts. B-D) Biomarker mRNA expression is represented on the y-axis while the frataxin mRNA expression is represented on the x-axis. Correlation was identified as strong if the coefficient is ±0.8, somewhat strong at ±0.7 and moderate at ±0.5 (n = 20, p<0.05*, p<0.01**, p<0.001***). Data in S4 File.

When FA b-lymphoblasts were treated with DMF for 48 hours *in vitro*, frataxin expression was increased 2.56 fold at the highest dose (p<0.00092, n = 6) and showed dose-dependent stimulation of frataxin expression at 3μM, 10μM and 30μM DMF (Fig 6A). At the mRNA level, two biomarker candidate genes: NCF2 and PDLIM1 showed altered expression in response to the DMF exposure, with a high correlation coefficient to relative frataxin mRNA expression.

In vehicle-treated FA lymphoblasts, NCF2 and PDLIM1 were 0.42 fold (p<0.0000021, n = 6) and 1.88 fold (p<0.000051, n = 6) relative to vehicle-treated healthy control, respectively (Fig 6A). By contrast, 30μM DMF dosing increased NCF2 2.41 fold (p<0.000093, n = 6) and decreased PDLIM1 0.50 fold (p<0.000030, n = 6) relative to vehicle-treated FA cells. At this dose, NCF2 and PDLIM1 were 1.02 fold (p<0.85, n = 6) and 0.94 fold (p<0.067, n = 6) respectively as compared to healthy control expression when normalizing the expression of these two biomarkers within control levels. However, the biomarker PRDX2 mRNA expression did not change treated with DMF (Fig 6A).

Correlation analysis was performed to elucidate the dependence of these biomarkers to frataxin abundance. In concurrence with the second qPCR screening of biomarker candidates, NCF2 showed strong positive correlation with a coefficient of 0.82 (p<0.0001, n = 20), and PDLIM1 showed moderate negative correlation to frataxin expression with correlation coefficient of -0.57 (p<0.0033, n = 20) (Fig 6B and 6C). In agreement with its response to DMF treatment, PRDX2 showed no correlation to frataxin expression (Fig 6D). To prevent bimodal frataxin expression driving the correlation, healthy control data was removed from the correlative analysis. Taken together, of the four biomarker candidates that had passed the primary qPCR array, secondary in-house qPCR analysis, and tertiary protein screen, only two biomarkers, NCF2 and PDLIM1, showed a consistent frataxin-dependent response at the mRNA level. Furthermore, the mRNA expression of the candidate biomarkers NCF2 and PDLIM1 was significantly altered in FA cells, and at the highest dose of DMF was observed to revert back to healthy control levels with no significant difference in expression level.

#### Type 1 Histone deacetylase inhibitor (RGFP109/RG2833)

**Fig 7:**
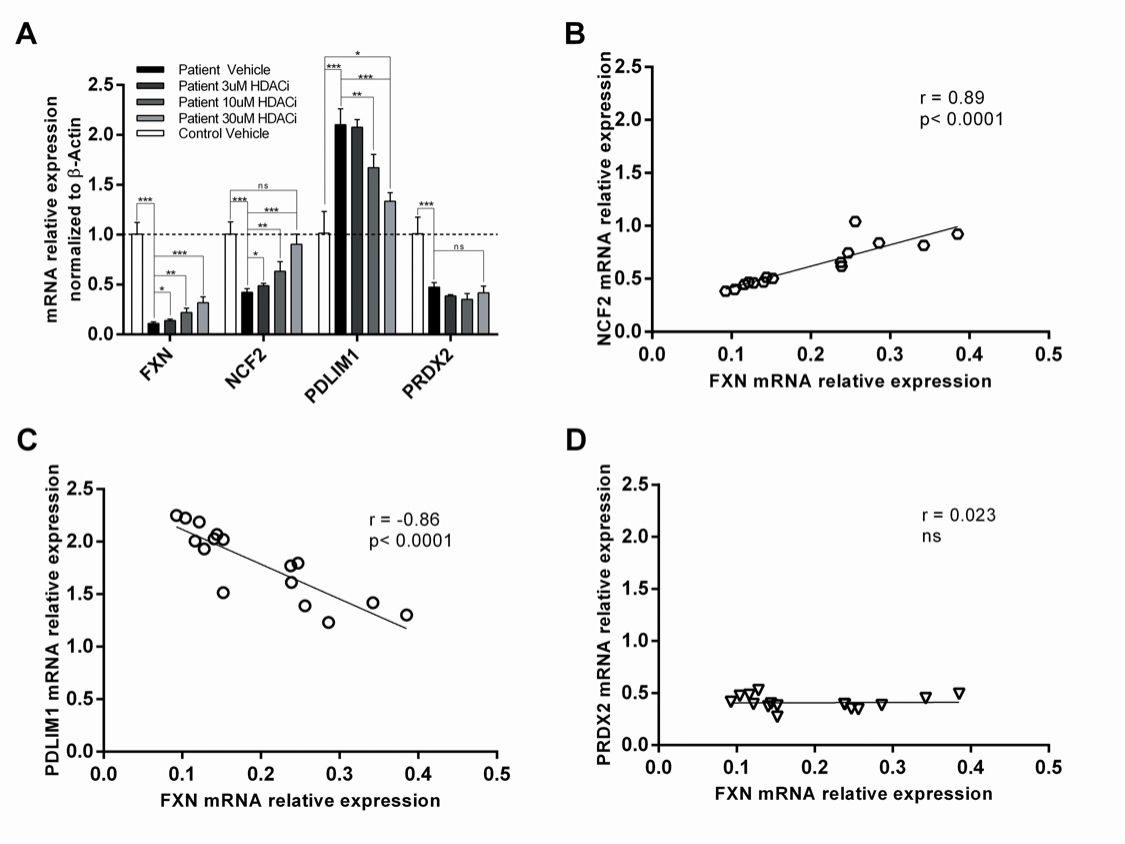
Friedreich’s ataxia biomarkers in response to 48 hours type 1 HDACi treatment of patient b-lymphoblast as compared to healthy control. A) Relative mRNA expression was normalized to β-actin using ΔΔ^CT^ calculation. The average expression of frataxin at vehicle, 3μM, 10μM, and 30μM HDACi is 0.11, 0.14, 0.22 and 0.32 relative to healthy control b-lymphoblasts, respectively. In the same order, PDLIM expression is 2.07, 2.04, 1.65 and 1.31 on average, NCF2 expression is 0.42, 0.49, 0.62 and 0.90 on average, and PRDX2 is 0.47, 0.38, 0.35 and 0.41 on average relative to healthy control b-lymphoblasts. B-D) Biomarker expression is represented on the y-axis while the frataxin expression is represented on the x-axis. Correlation was identified as strong if the coefficient is ±0.8 and somewhat strong at ±0.7 (n = 20, p<0.05*, p<0.01**, p<0.001***). Data in S5 File.

Another experimental Friedreich’s ataxia therapy, RGFP109/RG2833, was used to treat FA b-lymphoblasts for 48 hours *in vitro*. HDACi induced a dose-dependent unsilencing of frataxin expression at 3μM, 10μM and 30μM HDACi, and frataxin mRNA expression was increased 2.88 fold at the highest dose (p<0.00044, n = 4) relative to vehicle-treated FA cells (Fig 7A).

Initially, NCF2 and PDLIM1 were expressed 0.42 fold (p<0.00010, n = 4) and 2.069 fold (p<0.00019) in vehicle-treated FA cells relative to vehicle-treated healthy controls, respectively (Fig 7A). However, HDACi dose-dependently reversed the biomarker expressions of NCF2 and PDLIM1. At the highest dose of HDACi (30μM), expression of NCF2 was elevated 2.13 fold and PDLIM1 was decreased 0.63 fold to that of vehicle-treated FA cells (Fig 7A). Furthermore, the biomarker expressions were not significantly different from healthy control values.

Next, frataxin-dependency of the biomarker was tested by correlating biomarker expression with frataxin mRNA expression in HDACi treated cells. In agreement with the secondary qPCR screen and DMF dosing results, the biomarker NCF2 showed strong positive correlation to frataxin expression with a correlation coefficient of 0.829 (p<0.0001, n = 16), and PDLIM1 showed strong negative correlation to frataxin expression with a correlation coefficient of -0.86 (p<0.0001, n = 16). Similarly to the DMF dosing results, PRDX2 showed no correlation to frataxin expression in response to the HDACi treatment (Fig 7B–7D). Taken together, from the refined list of biomarker candidates and in concurrence with DMF dosing results, two biomarkers, NCF2 and PDLIM1, showed the highest correlation to frataxin expression and reverted to healthy levels in response to frataxin induction by HDACi in FA cells.

## Discussion

There is no current approved therapy for FA. One of the difficult issues in clinical drug development for FA is that all of the biomarkers are neurologic, including hole peg test, timed 25 foot walk, etc., and the uniformity of raters and scorers is important. The development of easily accessible, minimally invasive, rapidly quantifiable and scalar biomarkers of disease severity and/or drug effect would be a major support and accessory to clinical trials of drug effectiveness in FA. The benefit of the biomarkers NCF2 and PDLIM1 are that they correlate with frataxin expression and show changes in downstream effects of frataxin perturbation. We report here the identification of two biomarkers, NCF2 and PDLIM1 that have consistently different expression in FA and control comparisons, are correlated with frataxin expression, and are rescued back to normal control values by experimental FA therapies. The biomarkers in conjunction with frataxin expression aim to capture the drugs that restore frataxin expression, such as the HDACi RGFP109/RG2833 and the Nrf2 inducer DMF[29], or reverse the downstream pathomechanism of frataxin deficiency such as interferon gamma-1b[30] and the antioxidant EPI-743[31]. Many of these drugs are currently in clinical trials.

**Fig 8:**
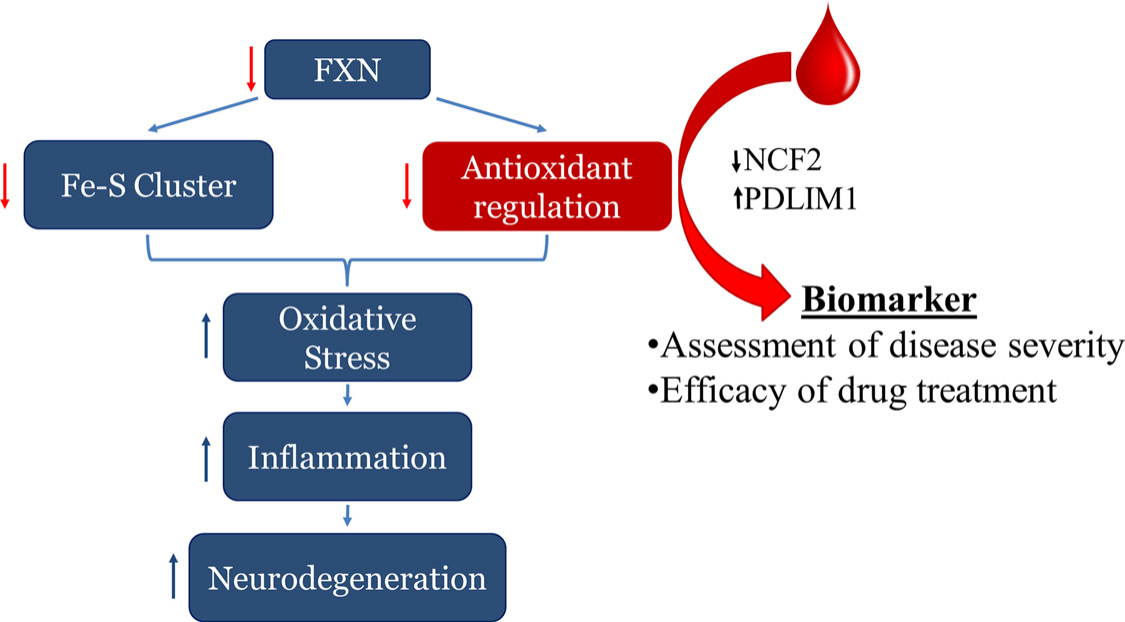
Ideogram for use of blood biomarkers to assess the Friedreich’s ataxia biomarker based on altered oxidative stress response gene expression. Whole blood or peripheral blood monocytes can be isolated from patients, and biomarker mRNA expression can be tested by qPCR techniques to biochemically assess disease severity and/or response to therapies.

The mechanism by which frataxin deficiency leads to elevated ROS and dysregulation of oxidative stress responses are not clearly understood but have been observed in FA patients, patient-derived cell lines and various FA animal models[17, 18, 35, 36]. Furthermore, the reduction of antioxidative capacity in FA cells is believed to contribute to the pathogenesis of this disease state, where the pathway is comprised of superoxide dismutase, Nrf2 regulated genes (NQO1 and HO1), and those mediated by a thiol group such as glutathione, thioredoxin and peroxiredoxin[23–26]. Aside from measuring frataxin expression, there are no robust, scalar, molecular biomarkers known to be related to Friedreich’s ataxia disease severity and progression. We suggest that lymphoblast mRNA levels of NCF2 and PDLIM1 could potentially be used as supplementary biomarkers of FA in addition to frataxin expression (Fig 8). These biomarkers are beneficial because they not only associate with frataxin expression but can identify if a drug rescues the downstream oxidative stress response associated with FA. Furthermore, we suggest the use of qPCR-based mRNA quantification compared to protein as a measurement of biomarker expression, as these data show a more robust correlative relationship between the biomarkers and frataxin expression in b-lymphoblasts compared to measuring protein abundance by western blot (Figs 3 and 5). This result is likely due to the higher precision and accuracy of qPCR transcript quantification compared to protein quantification by western blot procedure.

The primary and secondary screening of biomarker mRNA expression combined with the tertiary screening of biomarker protein level identified four candidate biomarkers out of the 84 original genes. When tested for their response to DMF and HDACi treatments, two final biomarkers became the most likely candidates for future therapeutic purposes. These two biomarkers, NCF2 and PDLIM1, were consistently altered in FA patient-derived cells as compared to healthy control b-lymphoblasts (Figs 1, 2 and 4). NCF2 is a subunit of the NADPH oxidase complex responsible for the synthesis of superoxide in neutrophils[37]. Possibly because of NCF2's pro-oxidant nature, NCF2 is suppressed in FA lymphoblasts[38].

PDLIM1 is also known as CLP36. Little is known about the role of PDLIM1/CLP36 in oxidant stress, however it was recently demonstrated to be a biomarker of the Nrf2 inducer sulforaphane in cardiomyocytes[39]. The major cause of death in Friedreich's is a cardiomyopathy that has some features of an inflammatory cardiomyopathy. Because PDLIM1/CLP36 is regulated by Nrf2/antioxidant status in cardiomyocytes[39] and it lymphoblasts (Fig 6), the PDLIM1/CLP36 status in peripheral blood lymphocytes could be considered a biomarker of cardiac antioxidant status. PDLIM1/CLP36 has also been shown to interact directly with actinin in the dorsal root ganglion, modulating the regenerative process of peripheral motor neurons[40]. PRDX5 is a gene in the peroxiredoxin family not regulated by Nrf2 that has previously been shown to be down regulated in FA patients[23].

Noteworthy biomarker candidates that passed the primary qPCR array and secondary qPCR screening but were unresponsive to DMF and HDACi treatments are PRDX5 and SFTPD. PRDX5 is a mitochondrial antioxidant responsible for scavenging excess peroxides in the mitochondrial matrix that is protective from the formation of DNA lesion and apoptosis[41, 42]. Lastly, SFTPD is an oxidative stress response gene that has been shown to be upregulated when exposed to hydrogen peroxide and is capable of inducing the Nrf2 antioxidative pathway[43, 44].

The biomarker candidates NCF2 and PDLIM1 showed strong and somewhat strong correlation to relative frataxin expression in FA b-lymphoblasts (Figs 3 and 5). Interestingly, the correlation coefficients of the biomarkers were both positive and negative, allowing for the detection of false biomarkers signal during the analysis of their response to FA therapies DMF and HDACi.

We treated FA b-lymphoblasts with DMF and HDACi to understand whether the candidate biomarkers are applicable for monitoring the efficacy of FA therapeutics *in vitro*. We show here a DMF-dependent induction of frataxin expression in FA patient lymphoblasts, and we had previously shown that DMF increases Nrf2 transcription factor binding to the frataxin gene promoter, which leads to an increase in frataxin expression[29].

The type 1 HDACi RGFP109/RG2833 is thought to be targeting HDAC1 and HDAC3, the likes of which were previously shown to increase the expression of the frataxin gene in FA patient peripheral blood mononuclear cells[32]. A mechanism of HDACi-induced frataxin unsilencing could be preventing the deacetylation of H3K9, H4K5, H4K8, and H4K16[28].

Because NCF2 is down regulated and PDLIM1 is up regulated dose-dependently in response to frataxin expression in FA b-lymphoblasts and is rescued by HDACi and DMF exposure, this increases our confidence and extends the list of potential molecular, scalar, minimally invasive biomarkers beyond frataxin.

Summary and Prospects. From the 84 original biomarker candidates screened, six were confirmed at the mRNA level, and four of these genes showed similar trends at the protein level (Figs 1, 2 and 4), wherein they showed a strong to somewhat strong correlation with frataxin expression in FA cells (Figs 3 and 5). Among those four, the expression of two biomarkers, NCF2 and PDLIM1, was dose-dependently normalized by exposure to DMF and HDACi. DMF and HDACi are experimental therapeutics that unsilence frataxin expression. Thus NCF2 and PDLIM1 are the most robust biomarkers of the 84 because of 1) their strong to somewhat strong correlation to relative frataxin expression, 2) their bidirectional change in expression in response to frataxin induction that can be used to reduce false positive signals, and 3) their lack of co-regulation, providing two independent indications of frataxin reconstitution or disease amelioration. 4) Furthermore, because PDLIM1/CLP36 has been shown by others to be a biomarker of oxidant/Nrf2 status in cardiomyocytes, and because cardiomyopathy is the cause of death in 80% of Friedreich's cases, a marker of its status could be important for clinical drug development. In the future, the lymphoblast mRNA biomarkers might be paired with the Friedreich’s ataxia rating scale (FARS) to provide objective, quantitative, scalar and minimally invasive assessment of disease severity in FA patients.

The use of biomarkers can potentially increase the speed of discovery and development of FA therapeutics while providing a quantitative measurement of FA patient health. Further studies are necessary to understand if the biomarkers behave similarly in patients being treated with currently developing therapeutics and if the biomarkers correlate with FARS.

## Supporting Information

S1 File Primary screen qPCR. Alteration of mRNA level oxidative stress response genes.
(XLSX)

S2 File Secondary screen qPCR. In-house mRNA level confirmation of primary screen.
(XLSX)

S3 File Tertiary screen Western. Protein level confirmation of secondary screen.
(XLSX)

S4 File 48hr DMF response qPCR. NRF2 mediated frataxin amelioration leads to NCF2 and PDLIM1 response.
(XLSX)

S5 File 48hr RG2833 response qPCR. HDAC inhibitor mediated frataxin amelioration leads to NCF2 and PDLIM1 response.
(XLSX)
